# Brn2 Is a Transcription Factor Regulating Keratinocyte Differentiation with a Possible Role in the Pathogenesis of Lichen Planus

**DOI:** 10.1371/journal.pone.0013216

**Published:** 2010-10-12

**Authors:** Ge Shi, Kyung-Cheol Sohn, Dae-Kyoung Choi, Yu-Jin Kim, Seong-Jin Kim, Bai-Sheng Ou, Yong-Jun Piao, Young Ho Lee, Tae-Jin Yoon, Young Lee, Young-Joon Seo, Chang Deok Kim, Jeung-Hoon Lee

**Affiliations:** 1 Department of Dermatology and Research Institute for Medical Sciences, School of Medicine, Chungnam National University, Daejeon, Korea; 2 Department of Dermatology, School of Medicine, Chonnam National University, Gwangju, Korea; 3 Department of Dermatology, The First Affiliated Hospital, Guangxi Traditional Chinese Medical University, Nanning, China; 4 Department of Dermatology, First Affiliated Hospital of Dalian Medical University, Dalian, China; 5 Department of Anatomy, School of Medicine, Chungnam National University, Daejeon, Korea; 6 Department of Dermatology and Institute of Health Sciences, School of Medicine, Gyeongsang National University, Jinju, Korea; Ludwig-Maximilians-University, Germany

## Abstract

Terminal differentiation of skin keratinocytes is a vertically directed multi-step process that is tightly controlled by the sequential expression of a variety of genes. In this study, we investigated the role of the POU domain-containing transcription factor Brn2 in keratinocyte differentiation. Immunohistochemical analysis showed that Brn2 is expressed primarily in the upper granular layer. Consistent with its epidermal localization, Brn2 expression was highly induced at 14 days after calcium treatment of cultured normal human epidermal keratinocytes. When Brn2 was overexpressed by adenoviral transduction, Brn2 led to increased expression of the differentiation-related genes involucrin, filaggrin, and loricrin in addition to inhibition of their proliferation. Chromatin immunoprecipitation demonstrated that Brn2 bound to the promoter regions of these differentiation-related genes. We injected the purified Brn2 adenovirus into rat skin, which led to a thickened epidermis with increased amounts of differentiation related markers. The histopathologic features of adenovirus-Brn2 injected skin tissues looked similar to the features of lichen planus, a human skin disease showing chronic inflammation and well-differentiated epidermal changes. Moreover, Brn2 is shown to be expressed in almost all cell nuclei of the thickened epidermis of lichen planus, and Brn2 also attracts T lymphocytes. Our results demonstrate that Brn2 is probably a transcriptional factor playing an important role in keratinocyte differentiation and probably also in the pathogenesis of lichen planus lesions.

## Introduction

Terminal differentiation of skin keratinocytes, in which the transition from basal keratinocytes to corneocytes is occurred, is a complex process that requires the simultaneous activation and inactivation of a wide variety of genes that must be expressed at the correct time and in the correct location [1]. Some characteristic genes expressed at different stages of keratinocyte maturation, such as involucrin, loricrin and filaggrin, are well documented [2]. In addition, a number of ubiquitous transcription factors, such as AP1, Sp1, and the AP2 family members, are involved in regulating keratinocyte gene expression and differentiation [3]. Although the functional involvement of many transcription factors in keratinocyte differentiation has been known, however, it is not sufficient to understand the sophisticated regulatory mechanism underlying this process. In this study, we identify a POU domain-containing transcription factor Brn2 as an important regulator in keratinocyte differentiation.

POU domain proteins have been implicated in development, replication, growth and cell cycle arrest, and differentiation [4]–[6]. Especially, POU domain-containing transcription factor Brn2 (also called N-Oct3 and POU3F2) has been implicated in both neuronal differentiation and activation of the corticotrophin-releasing hormone gene [7]–[9]. Targeted disruption of the Brn2 gene in mice results in loss of specific neuronal lineages in the hypothalamus and consequent loss of the posterior pituitary gland [9], [10]. Brn2 negative mice, therefore, die within 10 days of birth, although the specific cause of death is not apparent. As for skin cells, evidence also implicates it in melanoma growth and survival. Brn2 is overexpressed in human melanoma cell lines compared to normal melanocytes [11], [12], and it appears to play a role in melanoma cell proliferation and tumorigenesis [13], [14]. In melanoma, Brn2 is a focus for convergence of the MAP kinase and Wnt/β-catenin signaling pathways that are linked to cell proliferation [15], [16]. However, the expression and putative role of Brn2 in keratinocytes have not been clearly elucidated yet.

Although the importance of Brn2 in neuronal differentiation and melanoma development is recognized, however, the expression and putative role of Brn2 in epidermal keratinocytes have not been clearly elucidated yet. In this study, we provide evidences that Brn2 is a transcriptional factor playing an important role in keratinocyte differentiation, and probably also in the pathogenesis of lichen planus lesions.

## Results

### Expression of Brn2 in epidermal keratinocytes

**Figure 1:**
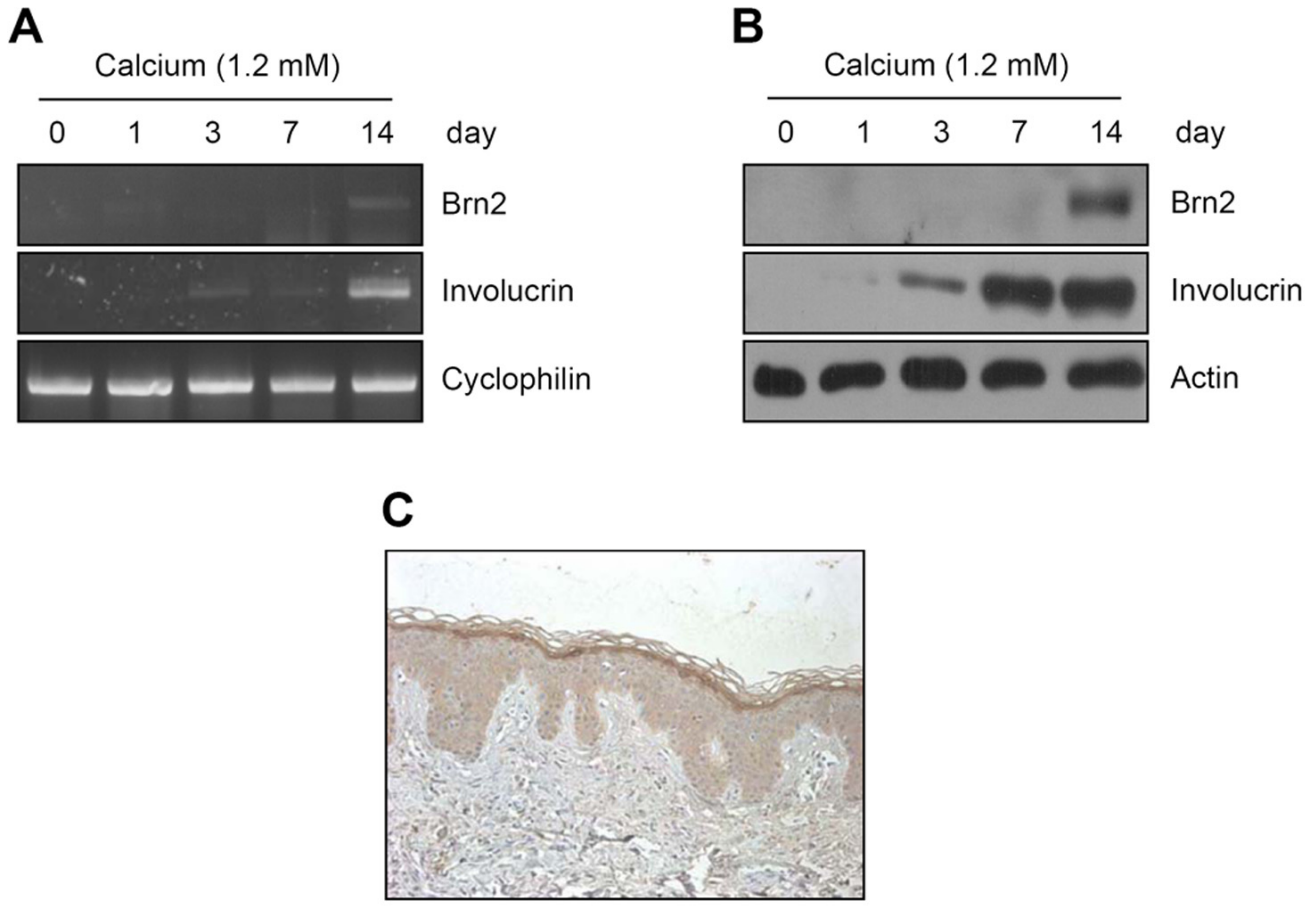
Expression of Brn2 in epidermal keratinocytes. (A) Keratinocytes were treated with 1.2 mM calcium at the indicated time points. The mRNA level for Brn2 was increased in a time-dependent manner during calcium-induced keratinocyte differentiation. Involucrin was used as a positive control for induction of keratinocyte differentiation. Cyclophilin was used as a loading control. (B) The protein level for Brn2 was detected by Western blot analysis. Involucrin and actin were used for positive control and loading control, respectively. (C) Immunohistochemical analysis of the Brn2 expression. Section of paraffin-embedded skin tissue was stained with anti-Brn2 antibody. Brn2 expression was increased in the granular layer.

To investigate the Brn2 expression during keratinocyte differentiation, we adopted a well-established calcium-induced differentiation model [17]. RT-PCR analysis clearly showed that the expression of Brn2 was increased at 14 days after calcium treatment (Figure 1A). Protein analysis using Western blotting also identified endogenous Brn2 expression in 14 day induced cells (Figure 1B). Consistent with, immunohistochemistry showed that Brn2 expression was increased in the granular layer of the epidermis (Figure 1C).

### Brn2 regulates the expression of keratinocyte differentiation markers

**Figure 2:**
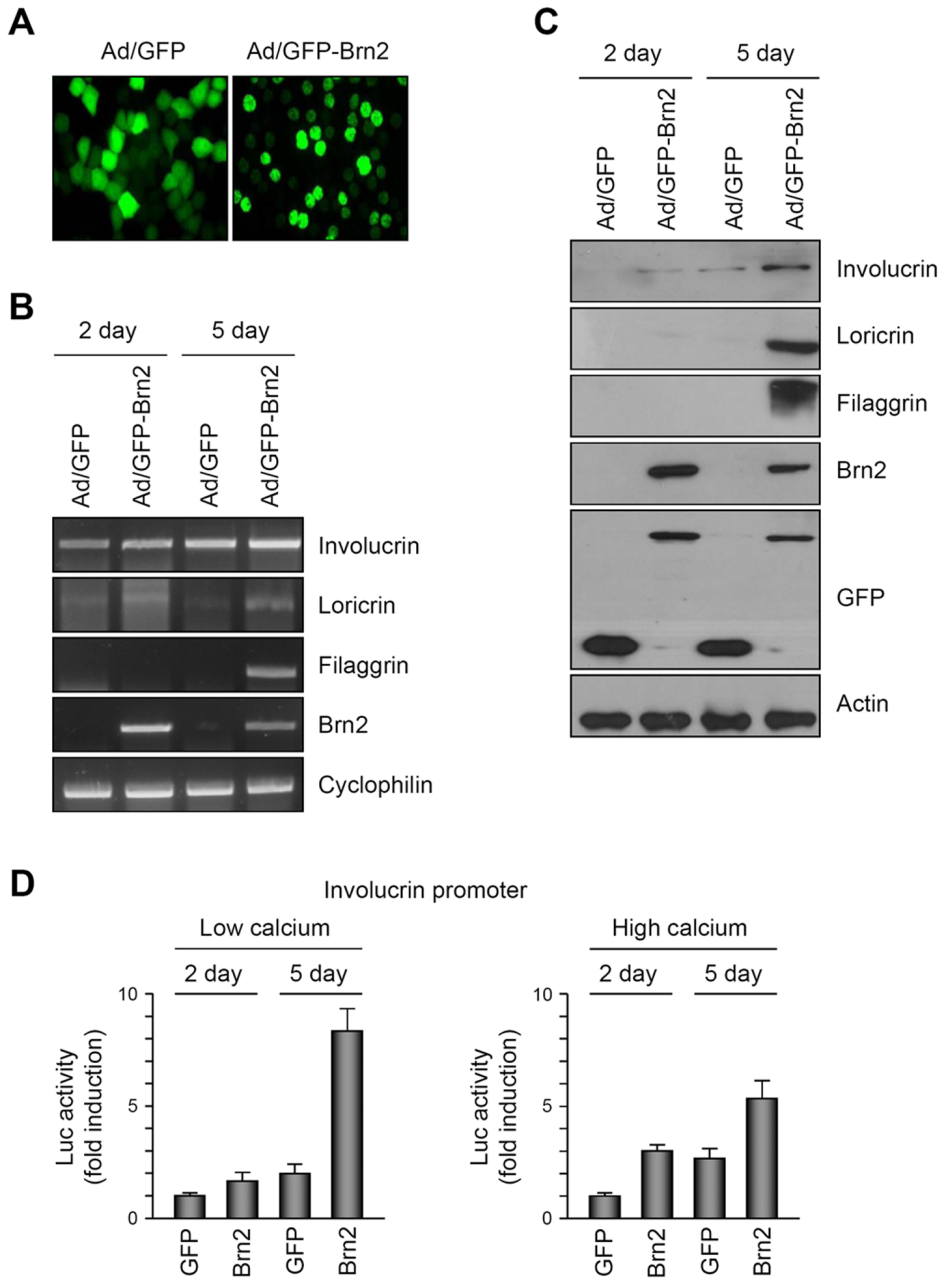
Keratinocyte differentiation by Brn2. (A) Expression of exogenous Brn2 in keratinocytes cultured in vitro. Cells were transduced with adenovirus expressing GFP-tagged PITX2c (GFP-PITX2c) at 10 multiplicity of infection (MOI) for overnight. Cells were replenished with fresh medium and incubated for 2 d. The expression of exogenous Brn2 was observed under the fluorescent microscopy. Adenovirus expressing GFP (Ad/GFP) was used as a negative control. (B) Effect of PITX2c on the expression of differentiation markers. Keratinocytes were transduced with adenovirus expressing GFP-Brn2 at the 10 MOI for overnight. Cells were replenished with fresh medium and incubated for the indicated time points. The mRNA level was verified by RT-PCR. Involucrin, loricrin and filaggrin are the markers for keratinocyte differentiation. (C) Western blot analysis. Cellular extracts were prepared after adenoviral transduction, and the protein level for differentiation markers was verified. In Western blot with anti-GFP antibody, upper bands indicate the GFP-fused Brn2 and lower bands indicate the GFP protein. (D) Effect of Brn2 on the involucrin promoter activity. Keratinocytes were transduced with 10 MOIs of involucrin-luc together with GFP-Brn2 expressing adenoviruses. Cells were lysed and assayed for luciferase activity. Data are represented as fold induction and SEM, measured from three independent experiments.

Since the expression of Brn2 was increased in the granular layer of the epidermis as well as in the differentiated keratinocytes by calcium, we speculated that Brn2 has a role for keratinocyte differentiation. To test this idea, we made the recombinant adenovirus expressing green fluorescent protein-tagged Brn2 (GFP-Brn2), and transduced cultured human epidermal keratinocytes. When overexpressed, Brn2 was located in nuclei of keratinocytes (Figure 2A). We then determined the effect of Brn2 on the expression of keratinocyte differentiation markers. As shown in Figure 2B, Brn2 increased expression of keratinocyte differentiation markers involucrin, loricrin, and filaggrin. The same results were obtained at protein levels (Figure 2C). To determine whether Brn2 effect was at the promoter level, we transduced keratinocytes with involucrin-luc reporter adenoviruses, in which about 3.7 kb of involucrin promoter fragment was fused to luciferase gene. Overexpression of Brn2 significantly increased the luciferase activities, in both the absence and presence of calcium (Figure 2D).

**Figure 3:**
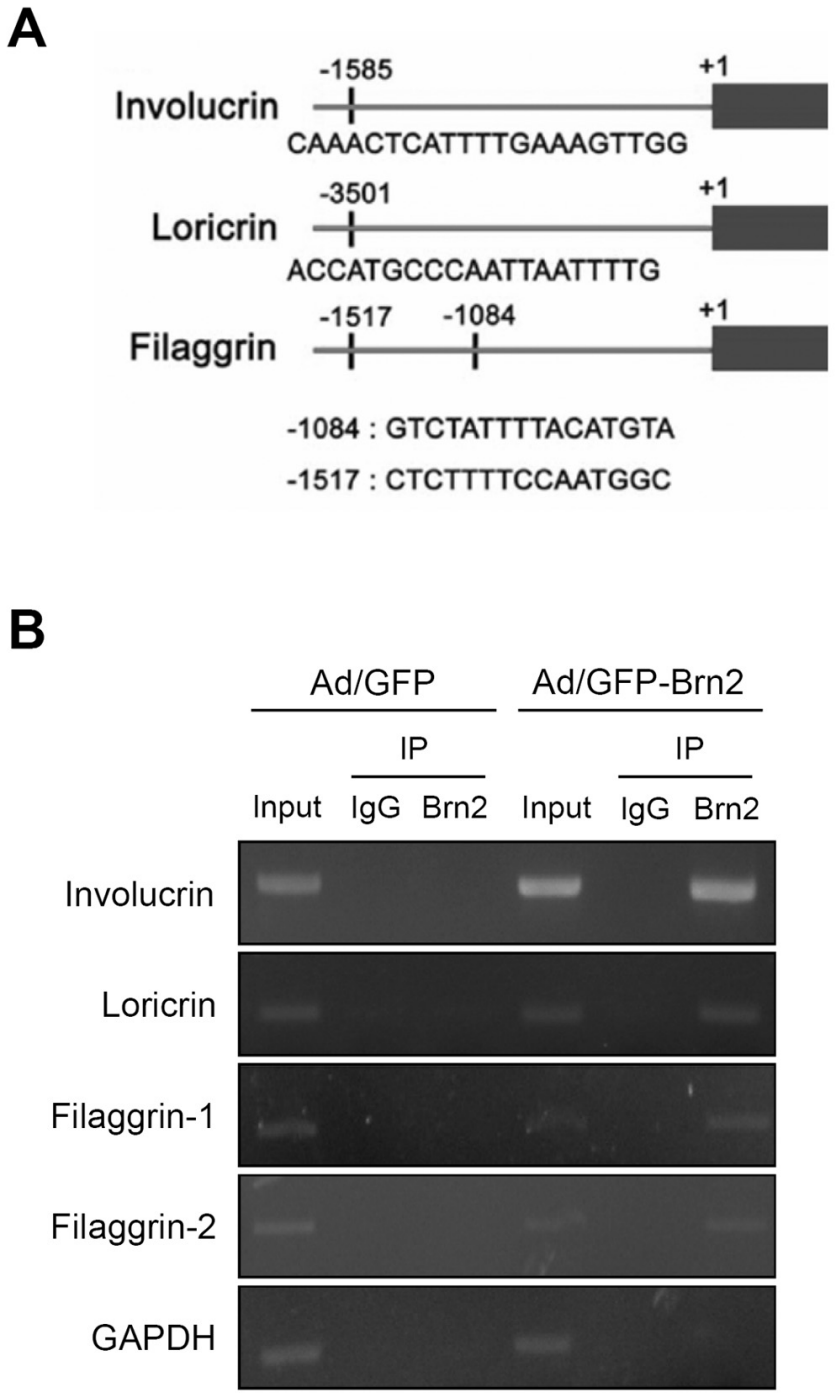
Chromatin Immunoprecipitation (ChIP) assay. (A) The putative binding sites for Brn2 were searched by in silico analytical method. (B) Keratinocytes were transduced with 10 MOIs of recombinant adenoviruses for overnight. Cells were replenished with fresh medium and cultured for 2 d. After cross-linking, nuclear fractions were isolated and immunoprecipitated with normal IgG or Brn2 antibody. Bindings of Brn2 to the promoters of involucrin, loricrin and filaggrin were verified by PCR. GAPDH was used as a negative control.

To further verify whether Brn2 directly regulates the transcription of differentiation-related genes, we searched for Brn2-binding site(s) using UCSC Genome Bioinformatics Program search engines and the Computational Biology Research Center program (http://genome.ucsc.edu.http://www.cbrc.jp/research/db/TFSEARCH.html). As a result, we found the putative Brn2-binding sites from promoters of involucrin, loricrin and filaggrin genes (Figure 3A). Consecutive chromatin immunoprecipitation (ChIP) assay clearly revealed that Brn2 bound to the promoters of involucrin, loricrin and filaggrin genes (Figure 3B). These results indicate that Brn2 is a functional transcription factor directly involved in the expression of involucrin, loricrin and filaggrin.

### Brn2 reduces the growth of keratinocytes

**Figure 4:**
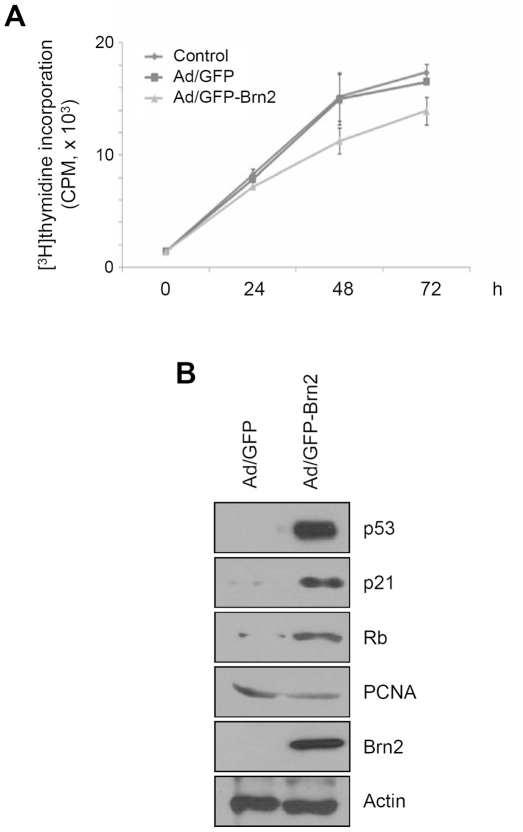
Effect of Brn2 on the growth of keratinocytes. (A) Keratinocytes were transduced with 10 MOI of adenovirus expressing GFP-Brn2 for overnight, washed twice with PBS, and incubated with fresh medium containing [^3^H]thymidine. At the indicated time points, cells were lysed and radioactivity was measured by scintillation counter. Control is non-virus transduced group. (B) Keratinocytes were transduced with adenovirus expressing GFP-Brn2 at the 10 MOI for overnight. Cells were replenished with fresh medium and incubated for 2 d. The protein level for cell cycle modulators were verified by Western blot.

Keratinocyte proliferation assays were performed to determine the effects of Brn2 on the keratinocyte growth. Keratinocytes were plated at a low density and after transduction with Brn2 adenovirus, grown for 3 days. The proliferation rate was determined using a thymidine assay. When Brn2 was overexpressed in keratinocytes, significant retardation of cell growth was observed (Figure 4A). To investigate how Brn2 affects the cell cycle, we determined which components of cell cycle-controlling molecules were affected by Brn2. As shown in Figure 4B, Brn2 increased the expression of the cell cycle regulators such as p53, p21 and Rb, while it decreased expression of PCNA.

### Brn2 increases the epidermal thickness and induces keratinocyte differentiation after intra-dermal injection into rat skin

**Figure 5:**
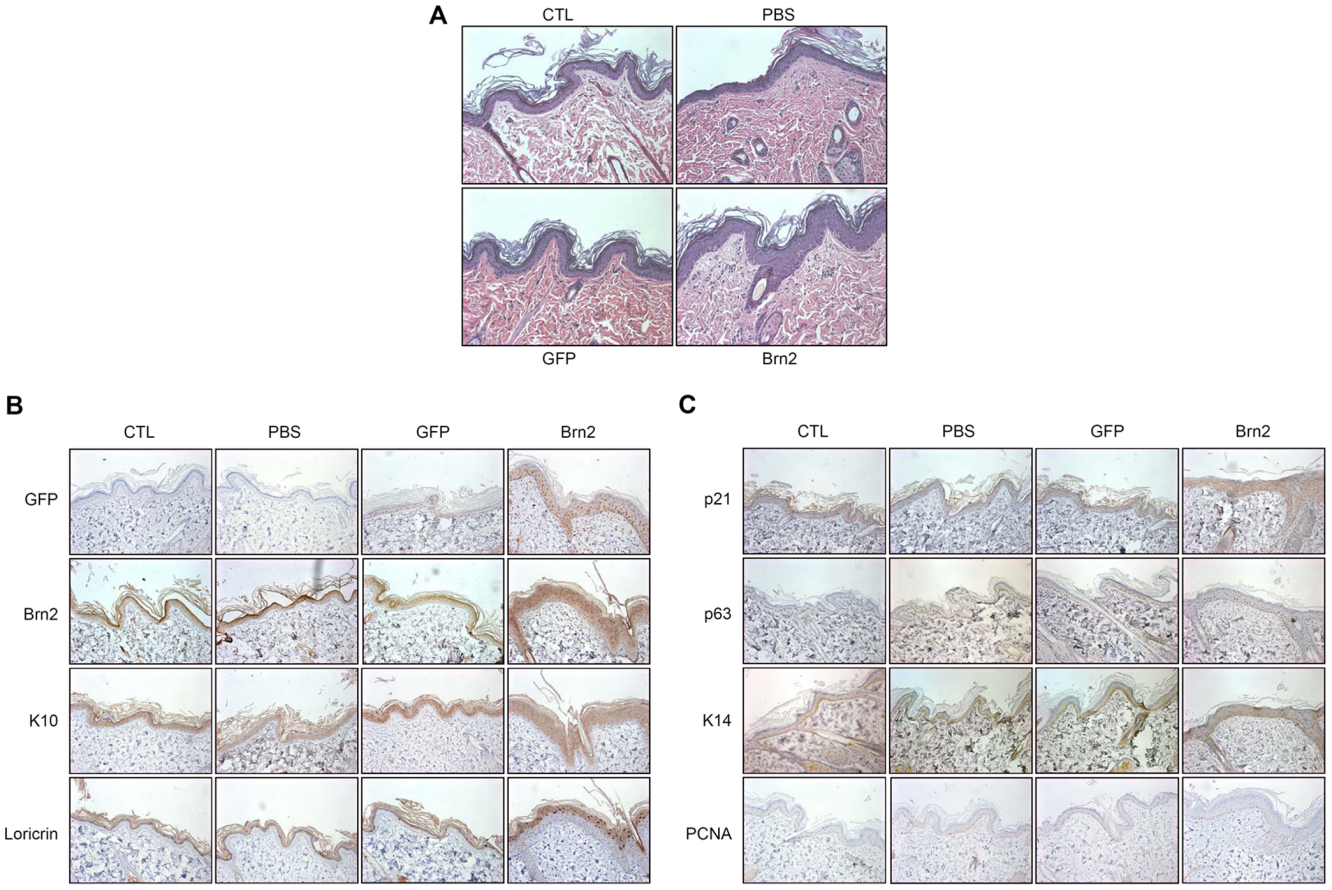
Ectopic expression of Brn2 in rat skin. (A) Female Sprague Dawley (SD) rats were intradermally injected with 50 µl of Brn2 expressing adenovirus (10^9^ particles). After 7 days, skin specimens were harvested and stained with hematoxylin and eosin. Epidermal thickness of Brn2-injected rat was greater relative to GFP-injected rat. CTL, non-injected; PBS, phosphate-buffered saline-injected. (B) Immunohistochemical staining of keratinocyte differentiation markers. Paraffin-embedded tissue sections were stained with anti-GFP, anti-Brn2, anti-keratin 10 (K10), and anti-loricrin antibodies. Expression of the early differentiation marker K10 and the late differentiation marker loricrin show that Brn2 can induces keratinocyte differentiation. (C) Immunohistochemical staining of proliferation-related genes. Sections were stained with anti-p21, anti-p63, anti-keratin 14 (K14), and anti-PCNA antibodies. The expression of p21 is increased in the Brn2-injected epidermis, there is no clear visible increase in K14 staining in the basal layer of the epidermis as in normal epidermis and there is not a large difference in the expression of p63 or PCNA between the control and the Brn2-injected epidermis.

We performed *in vivo* intra-dermal injection of the purified Brn2 adenovirus into rat skin. One week after injection, the thickness of the rat epidermis was increased to 6 to 8 cells, compared with 3–4 cells for uninjected skin or skin injected with either the PBS or GFP adenovirus (Figure 5A). Consistent with the predominant effects of Brn2 on the differentiation of cultured keratinocytes, epidermal layers expressing keratin10 and loricrin were increased by intra-dermal injection of Brn2 adenovirus (Figure 5B). Expression of keratin 14 was not significantly different between control- and Brn2-injected rat skin. There was no difference in the number of positive cells in the basal layer stained with the proliferation markers, anti-PCNA and anti-p63 antibodies. However, p21-expressing layers were significantly increased (Figure 5C). These results are not consistent with the decreased proliferation rate of keratinocytes transduced with Brn2 adenovirus (Figure 4A). Inconsistent results between *in vitro* cell cultures overexpressing Brn2 and *in vivo* data are thought due to other unknown *in vivo* factors that counteract the effect of Brn2.

### Brn2 intra-dermal injected rat skin is similar to lichen planus

**Figure 6:**
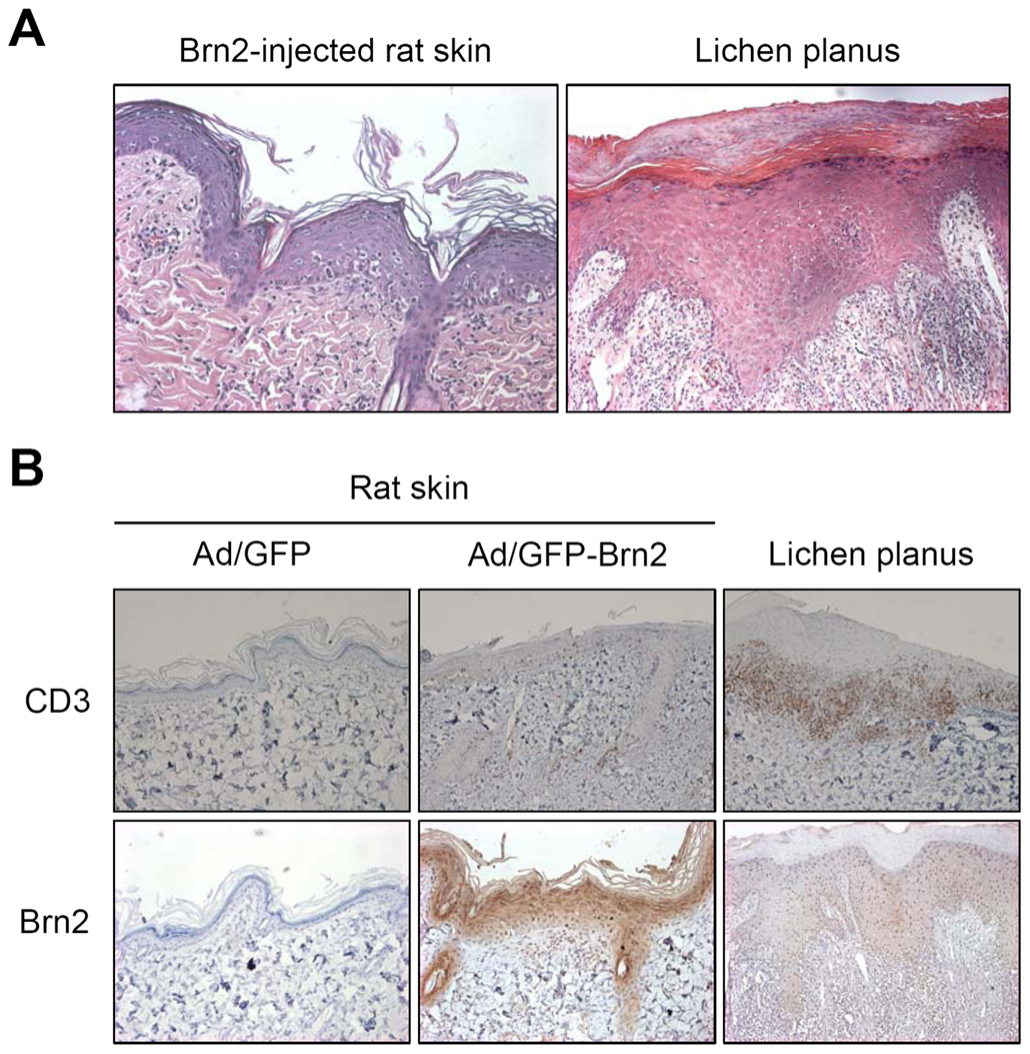
The histopathology of Brn2-injected epidermis is similar to human lichen planus. (A) In hematoxylin and eosin staining of Brn2-injected epidermis, hyperkeratosis with thickening of the granular cell layer, degeneration of the basal cell layer and infiltration of inflammatory cells into the subepithelial layer of connective are observed. (B) Immnunohistochemical analysis. T lymphocyte marker CD3 is increased in Brn2-injected rat skin and human lichen planus epidermis. Brn2 expression is increased in the nucleus of lichen planus epidermis.

**Figure 7:**
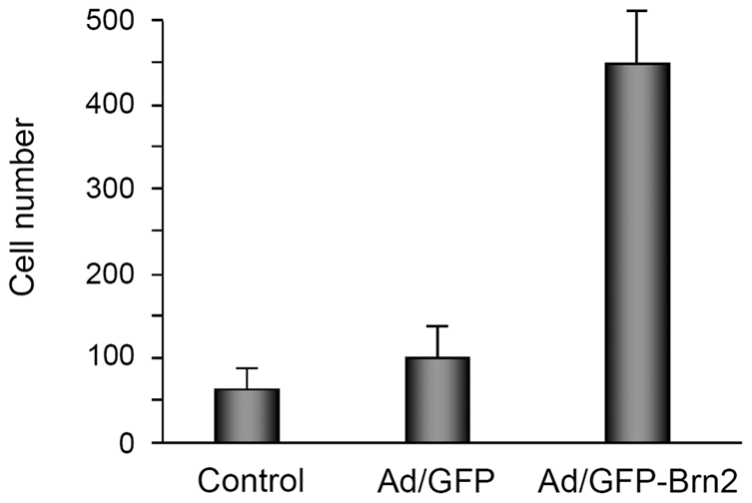
Brn2 induced chemotaxis of T lymphocyte migration. Jurkat cells were placed in upper well of Boyden's chamber. In lower well, keratinocytes were transduced with adenoviruses expressing GFP-Brn2 or GFP for overnight, replenished with fresh medium and incubated for 2 d. After assembly of upper and lower chambers, cells were further incubated contained keratinocytes transduced with the Brn2 and GFP adenoviruses for 48 h. After incubation for 24 h, the number of Jurkat cells that migrated across the filter was counted.

Although it was not all the case, the histopathology of Brn2 injected skin in some mice was similar to human lichen planus. Lichen planus is a well-known immunologically mediated skin reaction resulting in 1) hyperparakeratosis with thickening of the granular and spinous cell layers, 2) degeneration of the basal cell layer, and 3) infiltration of inflammatory cells into the subepithelial layer of connective tissue (Figure 6A). We examined the expression of Brn2 in the skin lesions of lichen planus and found that Brn2-expressing epidermal layers were highly increased in lichen planus compared to normal skin (Figure 6B). Lichen planus is a T-cell-mediated chronic inflammatory disease of unknown etiology. The lymphocytic infiltrates in lichen planus are composed almost exclusively of T-cells, and the majority of T-cells are activated CD3^+^ and CD8^+^ lymphocytes [18]–[21]. Consistent with this notion, CD3 positive T cells were infiltrated in the skin of the Brn2 adenovirus injected rat (Figure 6B). Thus, we hypothesized that Brn2-overexpressing keratinocytes may have impact on T cell migration. To test this idea, we used a chemotaxis assay with Jurkat T cells using a modified Boyden chamber analysis. Brn2-transduced keratinocytes attracted Jurkat T cells at about 6 fold greater than that of GFP-transduced keratinocytes (Figure 7). Thus, our results provide the first evidence that Brn2 has a role in the pathogenesis of lichen planus by promotion of T lymphocyte migration and by mediation of keratinocyte differentiation.

## Discussion

In this study, we demonstrated that Brn2 mRNA and protein levels were increased in 14 days after calcium treatment of primary keratinocytes, consistent with its exclusive expression in the granular layer of the normal epidermis. Using a recombinant adenovirus technique, we showed that Brn2 has a potential for promoting the keratinocyte differentiation. Actually, Brn2 induced the expression of differentiation markers such as involucrin, loricrin and filaggrin. These effects were believed to be a consequence of direct binding of Brn2 to the promoters of such genes, evidenced by ChIP assay. The most frequent targets of the POU domain proteins are the “octamer” motifs (ATGCAAAT), which are involved in both ubiquitous and cell-type specific regulation of various genes [11], [22]. These proteins also have the flexibility to bind heterogeneous sequences, such as the so-called TAATGARAT motif [22]. The proximal promoter region of the human profilaggrin gene contains two AT-rich motifs that are homologous to the consensus recognition sequence TAATGARAT of the POU domain transcription factors [23], [24]. In our study, the Brn2 binding motifs of the involucrin, loricrin and filaggrin promoter regions are not the same as the Oct binding motifs. However, they do have AT-rich motifs and nearly all binding sites have the same ATTTT motifs. Therefore, Brn2 increases differentiation of keratinocytes through increased transcriptional activity via specific DNA binding to the promoter regions of differentiation-related genes.

Despite its potential for promoting the keratinocyte differentiation, in our study, the epidermal thickness of Brn2-injected rat skin significantly increased with the increased differentiation in the spinous and granular layers. Brn2 markedly inhibited cell proliferation when overexpressed in cultured keratinocytes, however cell proliferation in the basal layer looked like unaffected by Brn2-injecton in rat skin. The discrepancy between in vitro and in vivo data regarding cell proliferation remains to be elucidated. Interestingly, in keap1-null mice, epidermal thickness is increased along with the increased differentiation marker expression [25]. These results suggest that epidermal thickness is determined by the balance of keratinocyte proliferation and differentiation, and it is likely that the impairment of the balance between proliferation and differentiation could lead to the thickening of epidermis.

Since intradermal injection of adenovirus can transduce almost all cells in the dermis including fibroblasts and possibly immune cells and endothelial cells, there can be paracrine effects by which the changes in the epidermis are happened. Actually, in our preliminary study, overexpression of Brn2 in fibroblasts resulted in increased expressions of IL-6 and IL-8 (Figure S1 and S2). Furthermore, the conditioned medium collected from Brn2-overexpressed fibroblasts induced the growth of keratinocytes as compared with the control conditioned medium (Figure S3). Thus, it is likely that epidermal thickening in Brn2-injected rat skin may be partly linked to the paracrine effects of neighboring cells.

The histopathology of Brn2-injected rat skin was similar to human lichen planus, showing hyperparakeratosis with thickening of the granular cell layer, degeneration of the basal cell layer, and infiltration of inflammatory cells into the subepithelial layer of connective tissue. We demonstrated the Brn2 is highly expressed in almost all epidermal cell nuclei in lichen planus using immunohistochemistry with the anti-Brn2 antibody. High levels of loricrin have been identified in the hypergranulotic and hyperorthokeratotic epidermis of lichen planus in a study exploring molecular alterations in keratinocyte differentiation in lichen planus [26]. Involucrin has also been identified as a diagnostic marker in oral lichenoid lesions based on observations of involucrin reactivity in the skin and in oral lichen planus [27], [28]. There are also reports of high p53 and p21 expression levels in oral lichen planus [29], [30]. We found that overexpression of Brn2 in keratinocytes changed the expressions of involucrin, loricrin, p53 and p21, supporting the notion that Brn2 is closely linked to the pathogenesis of lichen planus.

Lichen planus is a pruritic, papular eruption characterized by a violaceous color, polygonal shape, and, sometimes, a fine scale. It is most commonly found on the flexor surfaces of the upper extremities, on the genitalia, and on mucous membranes. Lichen planus skin lesions are thought to be an immunologically mediated reaction involving T-cells because lichen planus histology is characterized by a dense, subepithelial lympho-histiocytic infiltrate with an increased number of intra-epithelial lymphocytes [31]. As a consequence, degeneration of basal keratinocytes occurs forming colloid (Civatte, hyaline, cytoid) bodies that appear as homogenous eosinophilic globules [32]–[37]. Brn2 adenoviral injection of rat skin led to detection of CD3 positive lymphocytes in the epidermis, but not in GFP injected or normal control tissues. However, the density of inflammatory cells in Brn2 injected skin was not as high as in lichen planus, which is characterized by a band-like pattern of inflammatory cell infiltration in subepidermal areas, denying the role of Brn2 in the cause of lichen planus. Our observations of rat skin were made after a one time injection with the Brn2 adenovirus. It is possible that Brn2 could attract more inflammatory infiltrates, like in lichen planus, in a model in which Brn2 is continuously produced. It is also possible that inflammation is triggered by other mechanisms that induce Brn2 expression in keratinocytes. Such mechanisms may exaggerate inflammation while inducing terminal differentiation via positive feed back cycles.

The lymphocytic infiltrates of lichen planus are composed almost exclusively of T-cells with a high expression level of CD3^+^ on the cell surface [38]. While the majority of T-cells within the epithelium and adjacent to damaged basal keratinocytes is activated CD8^+^ lymphocytes with cytotoxic effects, most lymphocytes in the lamina propria are CD4^+^ helper T-cells lacking cytotoxic effects [18], [20], [39]. We also detected CD3 positive lymphocytes in Brn2 adenovirus injected rat skin, but not in GFP injected control tissues. Most inflammatory cells were observed in the upper dermis with a few approaching the epidermis. Some basal keratinocytes exhibited vacuolar changes, which probably indicates the beginning of lichen planus. Altogether, these results suggest that Brn2 may have a role for pathogenesis of lichen planus. The precise relationship between Brn2 and lichen planus, however, remains to be elucidated.

In conclusion, we provide evidence that Brn2 has a role in mediating keratinocyte differentiation, and is possibly linked to pathogenesis of lichen planus. Further research in this area should document what kinds of phenotypes would be followed in the absence of Brn2 expression in the epidermis.

## Materials and Methods

### Ethics Statement

All human skin samples were obtained under the written informed consent of donors, in accordance with the ethical committee approval process of the Institutional Review Board of Chungnam National University School of Medicine (permit number: 07-07, Development of skin specific microarray for gene analysis of Korean atopic patients). All animal tests were approved by the Institutional Review Board of Chungnam National University School of Medicine (permit number, CNUCOM-2007-14, Development of animal model for atopic dermatitis).

### Cell culture

Primary epidermal keratinocytes were cultured according to the method previously reported [40]. Keratinocytes were maintained in keratinocyte-serum free medium (K-SFM) supplemented with epidermal growth factor (EGF) and bovine pituitary extract (Gibco BRL, Rockville, MD). Jurkat T lymphoma cells were maintained in RPMI 1640 medium supplemented with 10% fetal bovine serum (FBS) (Gibco BRL).

### Immunohistochemical staining

Skin samples were fixed in 10% formalin for 24 h and embedded in paraffin. Sections of skin specimens were dewaxed, rehydrated, then washed three times with phosphate-buffered saline (PBS). After treatment with proteinase K (1 mg/ml) for 5 min at 37°C, sections were treated with H_2_O_2_ for 10 min at room temperature, placed in a blocking-solution (Dako, Carpinteria, CA) for 20 min, followed by reaction with the appropriate primary antibodies. Sections were incubated sequentially with peroxidase-conjugated secondary antibodies (Upstate, Lake Placid, NY) and visualized using a Chemmate Envision Detection Kit (Dako). Following antibodies were used in this study: Brn2, PCNA, GFP, p63, p21, loricrin, PCNA and CD3^+^ (SantaCruz Biotechnologies, Santa Cruz, CA); keratin 10, keratin 14 (Babco, Richmond, CA).

### Reverse transcription-polymerase chain reaction (RT-PCR)

Total RNAs were isolated from keratinocytes using Easy-blue RNA extraction kit (Intron, Daejeon, Korea). Two µg of total RNAs were reverse transcribed with moloney-murine leukaemia virus (M-MLV) reverse transcriptase (ELPIS Biotech, Daejeon, Korea). Aliquots of RT mixture were subjected to PCR cycles with appropriate primer sets. The sequences for primers were as follows: Brn2, 5′- GGAGTAGGGACACTCCACCA and 5′-CAGGAAGCTGCATTTTGTG; involucrin, 5′-CAAAGAACCTGGAGCAGGAG and 5′-CAGGGCTGGTTGAATGTCTT; loricrin, 5′-GTGGGAGCGTCAAGTACTCC and 5′-AGAGTAGCCGCAGACAGAGC; filaggrin, 5′-GGCACTCATCATGCAGAGAA and 5′- ATGGTGTCCTGACCCTCTTG; cyclophilin, 5′-CTCCTTTGAGCTGTTTGCAG and 5′-CACCACATGCTTGCCATCCA.

### Western blot analysis

Cells were lysed in Proprep solution (Intron). Total protein was measured using a Bradford protein assay kit (Bio-Rad Laboratories, Hercules, CA). Samples were run on SDS-polyacrylamide gels, transferred onto nitrocellulose membranes and incubated with appropriate antibodies. Blots were then incubated with peroxidase-conjugated secondary antibodies, visualized by enhanced chemiluminescence (Intron). The following primary antibodies were used in this study: involucrin, loricrin (Santa Cruz Biotechnologies); Rb, p53 (Cell Signaling Technology, Danvers, MA); actin (Sigma, St. Louis, MO).

### Adenovirus

Aliquot of RT mixture was subjected to PCR cycles with primer set for Brn2 (5′-CGCTACGGATCCATGGCGACCGCAGCGTCTAA and 5′-CGCTACGGATCC TCACTGGACGGGCGTCTGCA). The amplified full-length cDNA for PITX2c was subcloned into pENT/CMV-GFP vector that has attL sites for site-specific recombination with a Gateway destination vector. The replication-incompetent adenoviruses were created using Virapower adenovirus expression system (Invitrogen) according to the method previously described [41]. The adenovirus was purified with cesium chloride according to the method previously reported [42]. For creation of involucrin-luc reporter adenovirus, genomic DNA isolated from keratinocytes was used as a template for PCR. Primer sequences were as follows: involucrin promoter, 5′-CTCCATGTGTCATGGGATATG and 5′-TCAACCTGAAAGACAGAAGAG. The resultant PCR fragments cover from −2,467 to +1,239 base pairs of involucrin transcription site (http://www.ensembl.org/Multi/blastview).

### ChIP assay

Cells were grown to 50% confluency and then transduced with adenovirus expressing GFP-Brn2. After 2 day incubation, cells were cross-linked using 1% formaldehyde (Sigma) at 37°C for 10 min, rinsed two times with cold PBS, and then harvested in PBS containing protease inhibitors. ChIP assay was performed as previously described [43].

### Cell growth analysis

For determination of cell growth, [^3^H]thymidine uptake assay was performed. Keratinocytes cells were seeded in 60-mm culture dish, transduced with adenovirus for overnight. Cells were replenished with fresh medium containing 1 µCi of [^3^H]thymidine (Amersham, Buckinghamshire, UK). Following incubation for the indicated time point, cells were washed twice with PBS and incubated with 0.1 N NaOH at room temperature. Radioactivity in cell lysates was measured by liquid scintillation counter.

### Luciferase assay

Cells were grown at 50% confluency in a 12-well culture plate, then co-transduced with reporter adenovirus and Brn2 expressing adenovirus. After adenoviral transduction, cells were replenished with fresh medium. Cells were further incubated for 48 h, and then cellular extracts were prepared using cell lysis buffer. Luciferase activities were determined using Luciferase assay system (Promega, Madison, WI), according to the recommended protocol.

### Intra-dermal injection of adenovirus in rat skin

Female Sprague Dawley (SD) rats, each weighing approximately 200 g, were used (Orient Bio, Gapyung, Korea). Fifty µl of recombinant virus solution (10^9^ particles) prepared in PBS was injected intradermally into the dorsal skin of rats using a microsyringe with a 28-gauge hypodermic needle. The rats were sacrificed 7 day after intradermal injection and the dorsal skins were removed for histochemical analysis.

### Chemotaxis assay

Chemotaxis assays were performed using a modified Boyden's chamber (Neuroprobe Inc., Gaithersburg, MD), as previously described [44]. Briefly, Jurkat T cells were suspended in RPMI and 1.5×10^5^ of cells/ml was placed in the upper well of the chamber. Lower well contained adenovirally transduced keratinocytes. After incubation for 24 h at 37°C, non-migrated cells were discarded, and cells that migrated across the filter were counted.

## Supporting Information

Figure S1 Dermal fibroblasts were transduced with recombinant adenoviruses for 6 h. After replenishing with fresh growth medium (DMEM supplemented with 10% FBS), cells were further cultured for 2 d. Total RNAs were isolated and RT-PCR was performed. Overexpression of Brn2 in fibroblasts resulted in increased expressions of IL-6 and IL-8, while the expressions of IGF and TGF-β were reduced.
(0.65 MB TIF)

Figure S2 Dermal fibroblasts were transduced with the indicated MOIs (multiplicity of infection) of recombinant adenoviruses for 6 h. After replenishing with fresh growth medium (DMEM supplemented with 10% FBS), cells were further cultured for 2 d. Culture medium were collected and the secreted IL-8 was determined using ELISA kit (Human IL-8 CytoSetTM, Biosource, Camarillo, CA). Overexpression of Brn2 led to increase of IL-8 secretion. Statistical significance was set at *P<0.05.
(0.75 MB TIF)

Figure S3 Dermal fibroblasts were transduced with 10 MOIs of recombinant adenoviruses for 6 h. Cells were replenished with fresh growth medium (DMEM supplemented with 10% FBS), and incubated for 1 d. Cells were then washed twice with PBS then refed with KGM and incubated for a further 2 d. Culture medium were collected and centrifuged. Supernatants were collected (conditioned medium, CM), and added to the keratinocyte culture at the 50% concentration. Keratinocytes were further incubated in the presence of 1 mCi of [3H]thymidine (Amersham, Buckinghamshire, UK) for the indicated time points. Cells were washed twice with PBS and incubated with 0.1 N NaOH at room temperature. Radioactivity in cell lysates was measured by liquid scintillation counter. Statistical significance was set at *P<0.05.
(0.93 MB TIF)
