# Supplementary figures and images for: Brn2 Is a Transcription Factor Regulating Keratinocyte Differentiation with a Possible Role in the Pathogenesis of Lichen Planus
Source: PLoS One. 2010 Oct 12;5(10):e13216. doi: 10.1371/journal.pone.0013216 (PMC2953493; doi:10.1371/journal.pone.0013216)

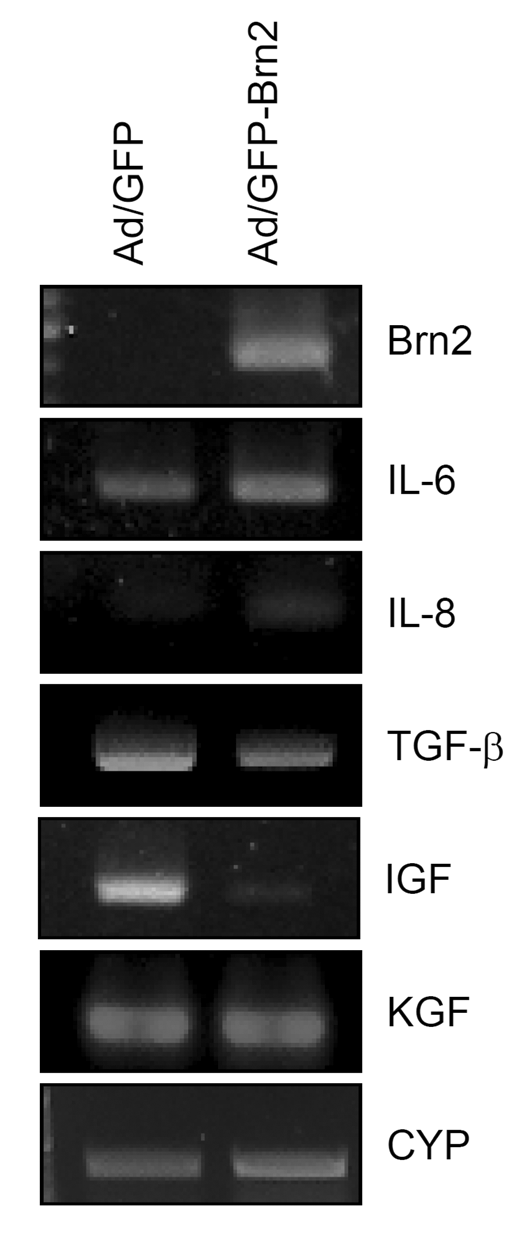

Supplement: Figure S1 — Dermal fibroblasts were transduced with recombinant adenoviruses for 6 h. After replenishing with fresh growth medium (DMEM supplemented with 10% FBS), cells were further cultured for 2 d. Total RNAs were isolated and RT-PCR was performed. Overexpression of Brn2 in fibroblasts resulted in increased expressions of IL-6 and IL-8, while the expressions of IGF and TGF-β were reduced. (0.65 MB TIF) [file pone.0013216.s001.tif]

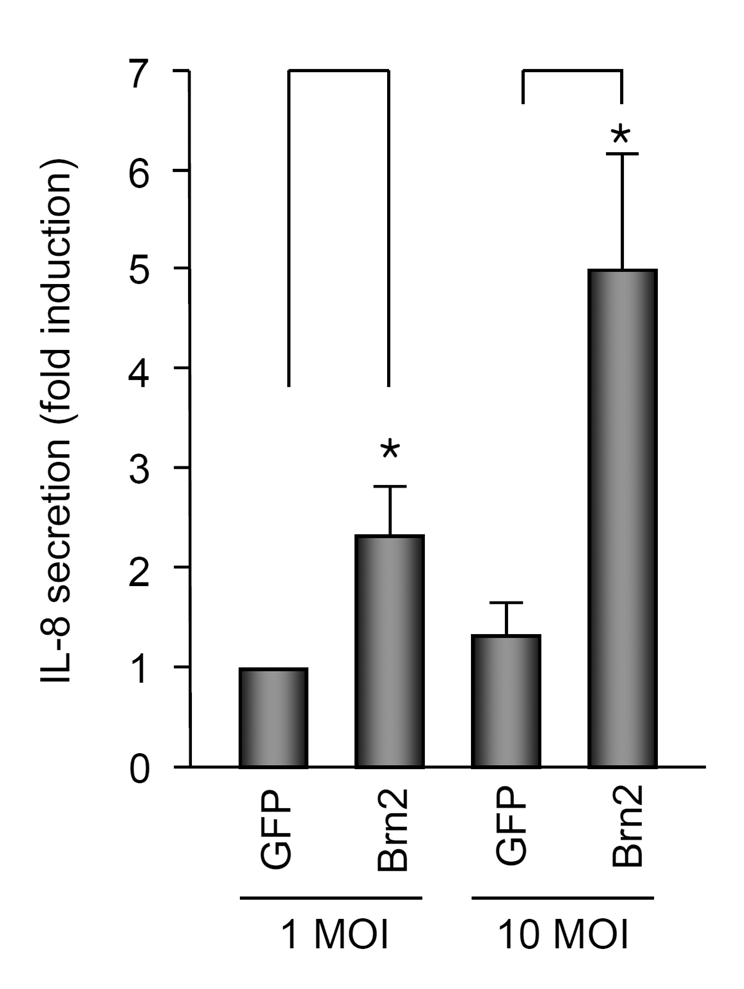

Supplement: Figure S2 — Dermal fibroblasts were transduced with the indicated MOIs (multiplicity of infection) of recombinant adenoviruses for 6 h. After replenishing with fresh growth medium (DMEM supplemented with 10% FBS), cells were further cultured for 2 d. Culture medium were collected and the secreted IL-8 was determined using ELISA kit (Human IL-8 CytoSetTM, Biosource, Camarillo, CA). Overexpression of Brn2 led to increase of IL-8 secretion. Statistical significance was set at *P<0.05. (0.75 MB TIF) [file pone.0013216.s002.tif]

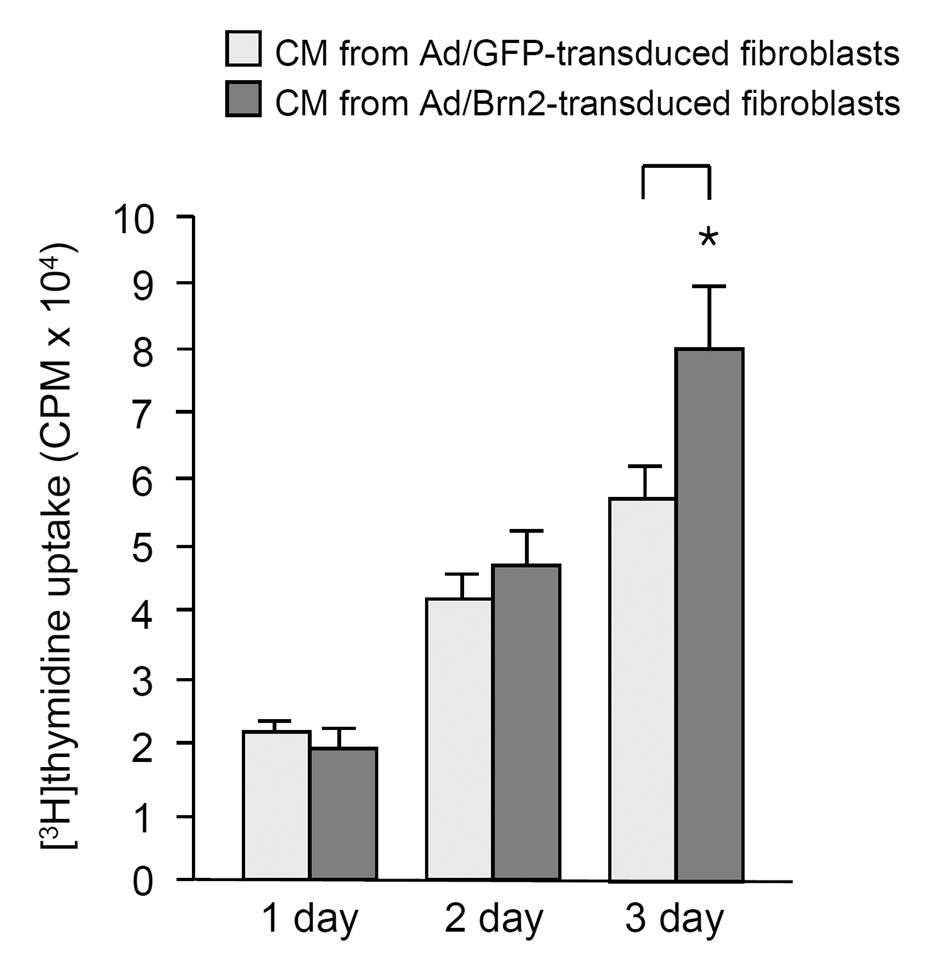

Supplement: Figure S3 — Dermal fibroblasts were transduced with 10 MOIs of recombinant adenoviruses for 6 h. Cells were replenished with fresh growth medium (DMEM supplemented with 10% FBS), and incubated for 1 d. Cells were then washed twice with PBS then refed with KGM and incubated for a further 2 d. Culture medium were collected and centrifuged. Supernatants were collected (conditioned medium, CM), and added to the keratinocyte culture at the 50% concentration. Keratinocytes were further incubated in the presence of 1 mCi of [3H]thymidine (Amersham, Buckinghamshire, UK) for the indicated time points. Cells were washed twice with PBS and incubated with 0.1 N NaOH at room temperature. Radioactivity in cell lysates was measured by liquid scintillation counter. Statistical significance was set at *P<0.05. (0.93 MB TIF) [file pone.0013216.s003.tif]
